# Functional Supramolecular Architectures of Dipyrrin Complexes

**DOI:** 10.3389/fchem.2018.00349

**Published:** 2018-08-15

**Authors:** Ryota Matsuoka, Tatsuya Nabeshima

**Affiliations:** Graduate School of Pure and Applied Sciences and Tsukuba Research Center for Energy Materials Science, University of Tsukuba, Tsukuba, Japan

**Keywords:** supramolecular chemistry, self-assembly, coordination bond, dynamic covalent bond, dipyrrin, BODIPY

## Abstract

Dynamic formation of self-assemblies from molecular components is a useful and efficient way to produce molecular and supramolecular architectures with sophisticated functions. The labile coordination bond and dynamic covalent bond as a reversible bond have often been used to create a well-organized supramolecular self-assembly. In order to realize sophisticated novel functions of the supramolecular self-assemblies, dipyrrin complexes have recently been employed as a functional unit and incorporated into the supramolecular architectures because of their outstanding properties and functions such as a high photostability and strong light absorption/emission. This review article summarizes recent development in functional supramolecular architectures of the dipyrrin complexes produced by coordination to a metal ion and dynamic covalent bond formation. We first describe the synthesis and unique functions of a series of discrete supramolecular architectures: helicates, macrocycles, and cages. The polymeric supramolecular self-assemblies with 1D, 2D, and 3D structures are then introduced as a functional infinite supramolecular architecture.

## Introduction

Dynamic formation of self-assemblies consisting of homo- and hetero-molecular components is a useful and efficient way to produce molecular and supramolecular architectures with sophisticated functions, which would not be realized by typical single molecules. The labile coordination bond and dynamic covalent bond (C = N bond, B–O bond, etc.) as a reversible bond have often been utilized to create a well-organized self-assembly at the molecular level (Holliday and Mirkin, 2001; Rowan et al., 2002; Alexeev et al., 2010; Jin et al., 2013). This reversible bond formation plays a very important role to easily prepare the desired most stable self-assembly because the most thermodynamically favorable structure is predominantly formed among the possible products which could be obtained from the starting materials. The dynamic chemical bonds can convert the kinetically-controlled products to the thermodynamically-stable ones, even if the unstable undesired products are first formed during the reaction.

The structure of the supramolecules is maintained by non-covalent bonds. In particular, a coordination bond to a metal ion has significant advantages over others because the defined bond direction and various valencies of the metal are available. Thus, we can design a variety of target supramolecular structures on the basis of the coordination bonds. One more important point of using the coordination bonds is that the metal complexes obtained by the coordination often provide a variety of properties and functions such as magnetism, redox activity, catalysts, luminescence, etc. Consequently, the incorporation of a metal complex into a supramolecular architecture should lead to highly functional molecular systems. In fact, many studies have already been reported regarding metallo-supramolecular systems (Balzani et al., 1998, 2008; Amendola et al., 2001; Collin et al., 2001; Sato et al., 2007; Brown et al., 2015). The combination of a ligand and metal ion for the coordination bond is also a key to selectively produce the supramolecular framework and functions.

**Figure 1:**
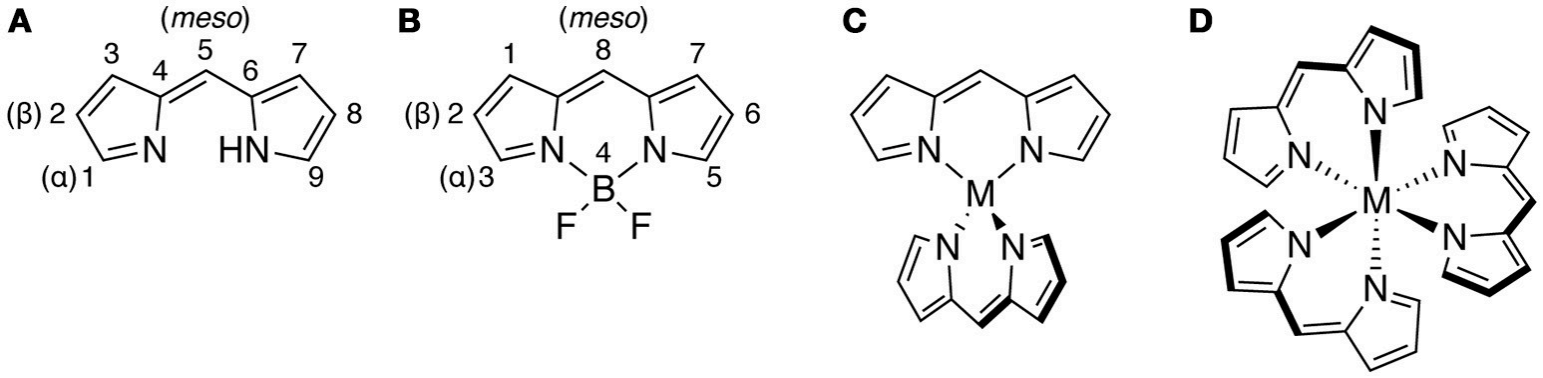
Chemical structures of **(A)** dipyrrin, **(B)** BODIPY, **(C)** bis(dipyrrinato)metal(II) complex, and **(D)** tris(dipyrrinato)metal(III) complex. The atomic numbering scheme is shown in **(A**,**B)**.

Recently, dipyrrin complexes have attracted considerable attention because of their unique properties (Wood and Thompson, 2007; Baudron, 2013; Nabeshima et al., 2015; Sakamoto et al., 2015b), which are different from those of other bidentate nitrogen ligands such as 2,2′-bipyridine etc. This is partly because the dipyrrins act as a monovalent ligand upon deprotonation to form a six-membered chelate ring with a transition metal or main-group element (Figure 1), while 2,2′-bipyridine, phenanthroline and α-imino pyridine are electrically neutral and bind to a metal ion to form a five-membered chelate ring. Although dipyrrin reacts with various elements to give the corresponding complexes, the boron complex of dipyrrin, BODIPY, has been most intensively investigated in many fields of science and technology because they are photochemically quite stable and usually show a sharp absorption and highly efficient luminescence (Loudet and Burgess, 2007; Ziessel et al., 2007; Benniston and Copley, 2009; Lu et al., 2014). In addition, the BODIPYs are applied to laser dyes, photovoltaics, electrochemical luminescent materials, bio-imaging, and photodynamic therapy (Ortiz et al., 2010; Benstead et al., 2011; Awuah and You, 2012; Boens et al., 2012; Kamkaew et al., 2013; Bessette and Hanan, 2014; Ni and Wu, 2014; Singh and Gayathri, 2014; Zhao et al., 2015; Antina et al., 2016). These attractive functions of BODIPY and other dipyrrin complexes encouraged us and other researchers to incorporate the dipyrrin complexes into the frameworks of the supramolecular self-assemblies (Shin et al., 2010; Antina et al., 2015).

We now introduce and review recent research regarding supramolecular architectures with the dipyrrin complexes produced by coordination to a metal ion and dynamic covalent bond formation. The discrete supramolecular systems, helicates, macrocycles, and cages are initially discussed. The polymeric supramolecular self-assemblies with 1D, 2D, and 3D structures are then summarized as an infinite supramolecular architecture.

## Self-assembly of discrete supramolecular architectures and their functions

Studies of discrete supramolecules with a well-defined structure are important and useful to deeply understand how the non-covalent interactions work to form supramolecules. These supramolecular architectures often provide valuable functions such as precise recognition, specificity in molecular conversion, and information storage. In this section, a series of discrete supramolecular architectures, helicates, macrocycles, and cages, based on the dipyrrin complexes are introduced.

### Self-assembled helical architectures

**Figure 2:**
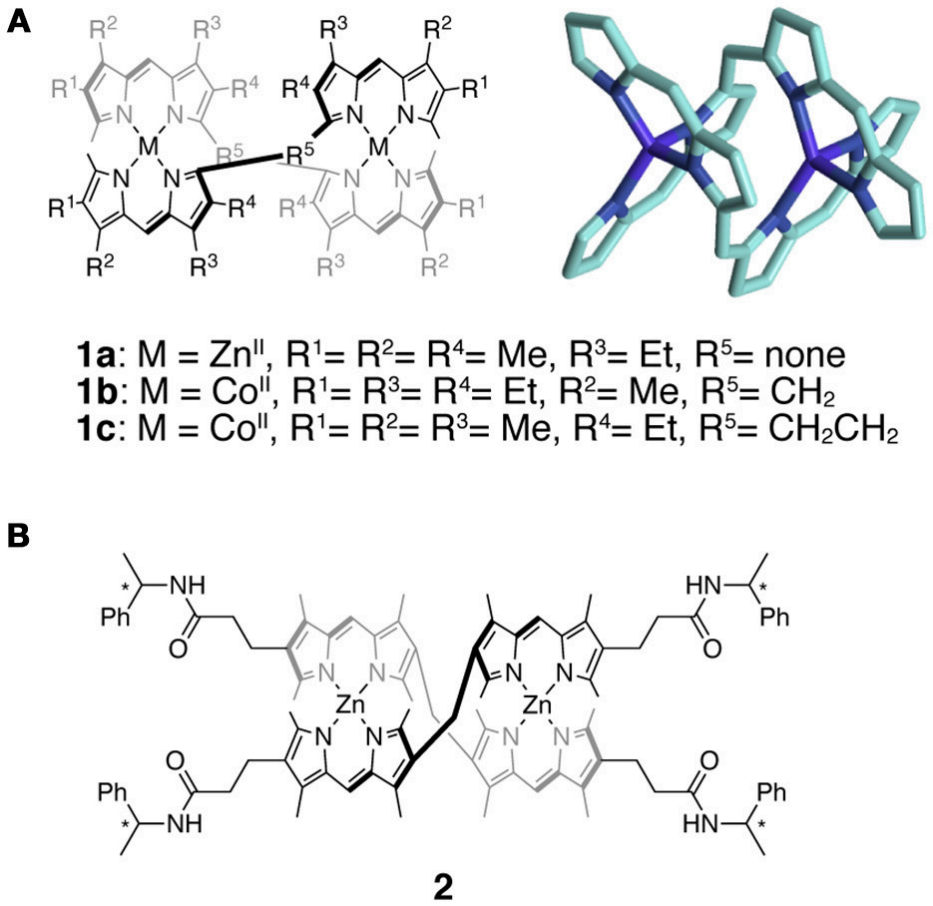
**(A)** (left) Chemical structures of double helicates **1a**–**1c**. (right) Molecular structure of **1b** determined by X-ray crystallography. Hydrogen atoms and peripheral methyl and ethyl substituents are omitted for clarity. **(B)** Chemical structure of the double helicate **2** bearing homochiral amide substituents.

Helical structures have inherent chiral information that plays important roles in biomolecular and synthetic supramolecular systems. In artificial systems, a representative example is the helicate, whose helical arrangement is held by coordination bonds. Studies of the double-stranded helicates based on the bis(dipyrrinato)metal(II) complexes were pioneered by Dolphin and coworkers. The helicates including **1a**–**c** (Figure 2A) were obtained by the self-assembly of bis- or tris(dipyrrin) ligands linked directly or by flexible spacers such as short alkyl chains (Zhang et al., 1998; Thompson and Dolphin, 2000a) and sulfur (Chen et al., 2002). To date, dipyrrin-based helicates have been developed to realize interesting functions and more elaborate structures (Bröring et al., 2007; Guseva et al., 2012). For example, several complexes containing Zn(II), Cd(II), or Hg(II) were reported to exhibit visible light emission (Yang et al., 2004; Antina et al., 2010, 2011a,b, 2013; Dudina et al., 2013, 2015; Bumagina et al., 2017). As another example, the diastereoselective synthesis of the bis(dipyrrinato)zinc(II) double helicate **2** was achieved by introducing chiral amide substituents at the termini of the bis(dipyrrin) ligand (Figure 2B) (Wood et al., 2005).

**Figure 3:**
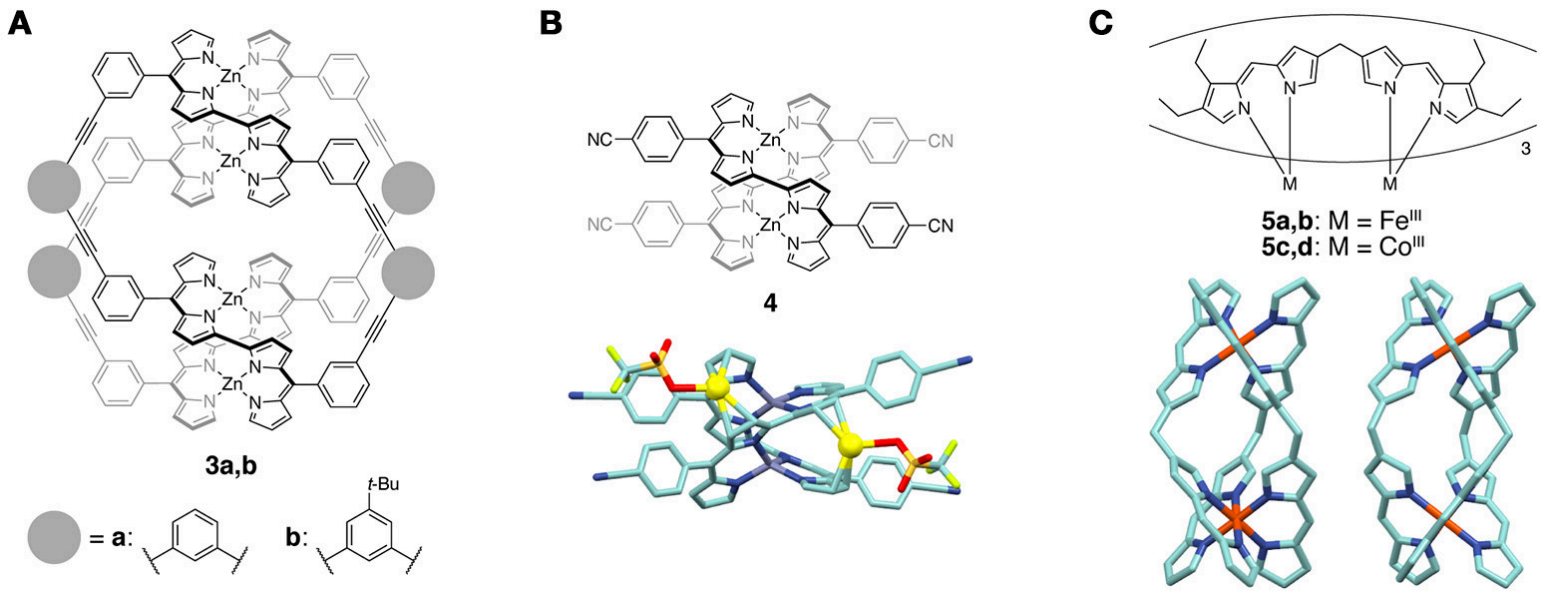
**(A)** Double helical complexes **3a**,**b** that show thermally responsive spring-like motions. **(B)** (top) Chemical structure of Zn(II) double helicate **4**. (bottom) Molecular structure of the complex of **4**, Ag(OTf), and THF determined by X-ray crystallography. THF and hydrogen atoms are omitted for clarity. The Ag(I) ions in the π-clefts of **4** are highlighted as yellow balls. **(C)** (top) Chemical structures of triple-stranded M_2_L_3_ complexes **5a**–**d**. (bottom) Molecular structure of helicate **5a** (left) and mesocate **5b** (right) determined by X-ray crystallography. Hydrogen atoms and peripheral ethyl substituents are omitted for clarity.

The structural change in the double helicates strongly affects the optical properties of the bis(dipyrrinato)metal(II) complexes. Maeda and coworkers covalently strapped two bis(dipyrrin) strands via the *meso*-aryl moieties to form macrocyclic structures (Figure 3A) (Hashimoto et al., 2010). This strapping significantly stabilized the Zn(II)-coordinated double helicates **3a** and **3b**. Optically-pure **3a** and **3b** were obtained by chiral HPLC, and the enantiomers of these helicates exhibit Cotton effects in the CD spectra. In addition, the complexes show thermally responsive spring-like motions because the dihedral angle between the dipyrrin moieties in each oligo(dipyrrin) ligand is flexible. This motion resulted in temperature-dependent changes in the UV-vis absorption, emission, CD, and NMR spectra. Similar strapped double helicates were then synthesized, and the exciton coupling between the multiple dipyrrin chromophores were observed (Maeda et al., 2013b). The structure of the double helicates was also controlled by steric or electrostatic repulsion between the terminal substituents at the α positions of the bis(dipyrrin) ligands (Maeda et al., 2014; Kong et al., 2017).

In some cases several unique cation-π interactions were observed on the aromatic rings of the dipyrrin-based double helicates. For example, a methylene-bridged bis(dipyrrinato)zinc(II) double helicate interacts with Ag(I) ions in the presence of the chiral lanthanide shift reagent probably due to Ag-π interactions (Thompson and Dolphin, 2000b). Later, the existence of interactions between Ag(I) ions and the π-surfaces of bis(dipyrrinato)zinc(II) helicates was proved in the solid state. The Zn(II) double helicate **4** prepared from 2,2′-bis(dipyrrin) ligands has a π-cleft structure consisting of pyrrol rings, which can bind to two Ag(I) ions by Ag-π interactions (Figure 3B) (Ruffin et al., 2014). The Ag(I) ions further interact with cyano groups in **4**, solvents, and/or counter anions to give self-assembled architectures with various dimensionality. Another interesting helical architecture is an anthracene-appended Zn(II)-bis(dipyrrin) helicate, whose anthracene moieties bind to Ag(I) ions by cation-π interactions (Baudron and Hosseini, 2016). Additional interactions between the Ag(I) ions and triflate anions give a 1D network structure in the solid state.

Triple-stranded helicates and mesocates based on octahedral coordination are also available when oligo(dipyrrin) ligands react with trivalent metal ions. An α-free bis(dipyrrin) ligand that has a methylene bridge at the β position coordinates to Co(III) or Fe(III) ions to produce two types of dinuclear triple-stranded M_2_L_3_ complexes **5a**–**d** (Figure 3C) (Zhang and Dolphin, 2009). NMR, MALDI-TOF-MS, and X-ray crystallographic analyses revealed that the two complexes are a homochiral helicate and achiral mesocate, which are not interconvertible even at elevated temperature. Similar results were also observed in the cases of the Ga(III) and In(III) ions (Zhang and Dolphin, 2010).

### Self-assembled macrocyclic architectures

Structural motifs of supramolecular architectures other than the helix are also available using dipyrrin complexes. The structure of the resultant assemblies strongly depends on the rigidity of the linkers tethering the dipyrrin units. In particular, the rigid linkage is often useful to prepare oligo(dipyrrin) molecular and supramolecular macrocycles.

**Figure 4:**
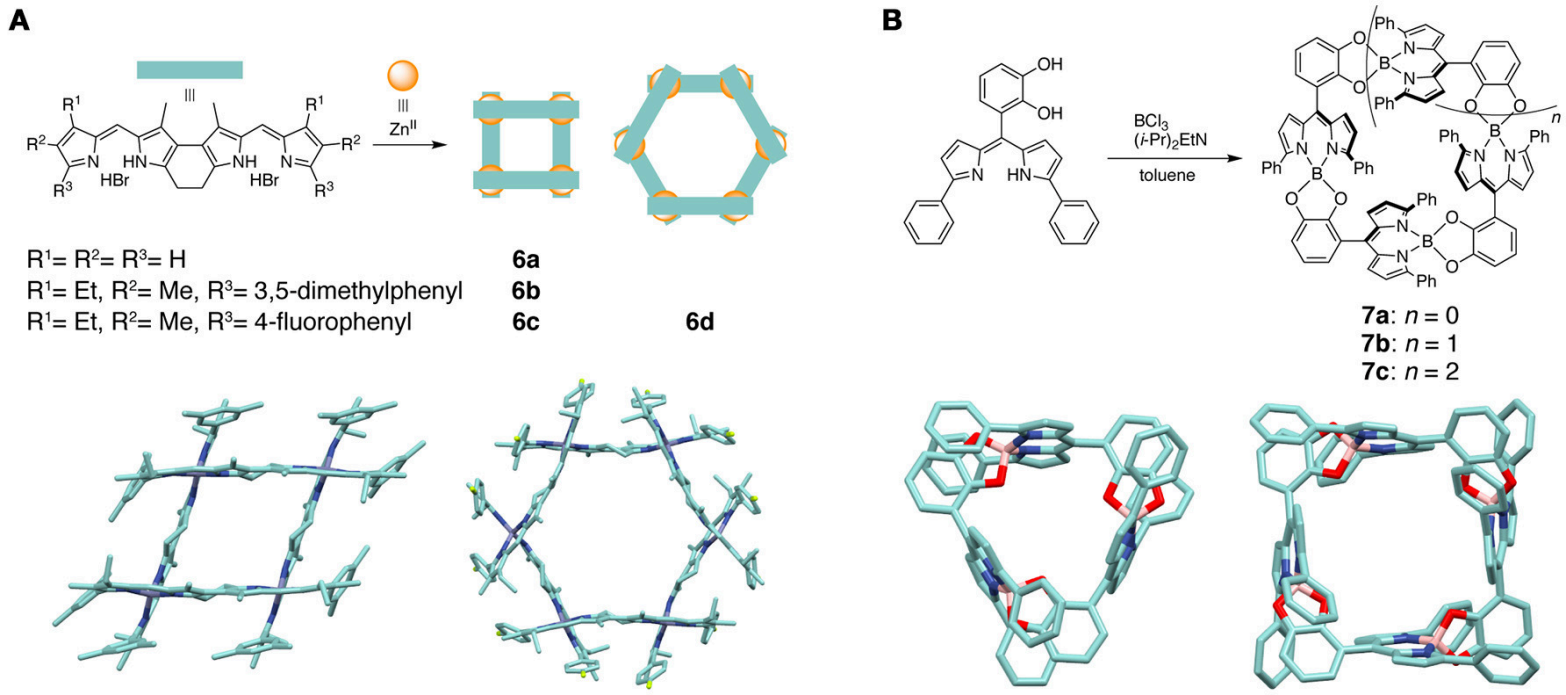
**(A)** (top) Schematic illustration showing the synthesis of grid-type rhombic (**6a**–**6c**) and hexagonal (**6d**) supramolecular macrocycles. (bottom) Molecular structure of **6b** (left) and **6d** (right) determined by X-ray crystallography. Hydrogen atoms are omitted for clarity. **(B)** (top) Synthetic scheme of macrocyclic complexes **7a**–**7c**. (bottom) Molecular structure of **7a** (left) and **7b** (right) determined by X-ray crystallography. Hydrogen atoms are omitted for clarity.

The supramolecular macrocycles based on the dipyrrin complexes are usually composed of bis(dipyrrinato)metal(II) complex units. Ligands comprising two dipyrrin units linked directly at the α or β positions were used to construct trimeric or tetrameric circular complexes (Thompson et al., 1999; Baudron et al., 2015). The bis(dipyrrin) ligands are arranged in the over and under fashion around the metal centers to form structures with a helicity (i.e., circular helicate). Alkyl chain spacers (*n* > 2) between the β positions inhibited the formation of macrocyclic structures, which then led instead to the monomeric or dimeric complexes. Ring-fused bis(dipyrrin) ligands were used to prepare grid-type rhombic and hexagonal supramolecular macrocycles **6a**–**6d** containing the bis(dipyrrinato)zinc(II) complex units (Figure 4A) (Ma et al., 2011). The ring-fused ligands are rigid enough to prefer the grid-type circular self-assembly upon coordination rather than the circular helicate formation. **6a**–**6d** have two intense absorption bands in chloroform, which are ascribed to the ligand-centered π-π^*^ transition and the metal-to-ligand charge transfer transition. Preparation of dinuclear and trinuclear bis(dipyrrinato)zinc(II) and nickel(II) macrocycles was achieved by tethering the *meso* positions of the two dipyrrins using a rigid phenyleneacetylene linker (Maeda and Hashimoto, 2007; Maeda et al., 2013a). Some of these complexes show a high hole mobility up to 0.11 cm^2^ V^−1^ s^−1^ in the crystalline state (by a non-contact flash-photolysis time-resolved microwave conductivity measurement), which originates from the well-aligned π-electron systems in close contact with each other.

The dipyrrin-based supramolecular macrocycles have been constructed not only from the homoleptic bis(dipyrrinato)metal(II) complexes, but also from heteroleptic mono(dipyrrinato) complexes of the main group and transition metal elements. Nabeshima and coworkers introduced a catecholyl group as the second coordinating site at the *meso* position of the dipyrrin ligand (Figure 4B) (Ikeda and Nabeshima, 2008). The boron complexation between the dipyrin and catecholyl moieties afforded the macrocyclic trimer **7a**, tetramer **7b**, and pentamer **7c** in which the (dipyrrinato)(catecholato)boron complex moieties are arranged in a head-to-tail fashion. Since the inner cavity of the trimeric macrocycle is surrounded by catecholate oxygens and electron-rich pyrrol planes, the macrocyclic complex **7a** strongly recognizes alkali-metal cations (K^+^, Rb^+^, and Cs^+^) in solution. Some other dipyrrin complexes of boron (Kaloudi-Chantzea et al., 2012; Martinou et al., 2017) and Zn(II) (Sutton et al., 2004), Ag(I) (Salazar-Mendoza et al., 2008; Pogozhev et al., 2011; Zhang et al., 2017a), and Re(I) (Zhang et al., 2017b) ions were also used as building blocks of the supramolecular macrocyclic architectures.

**Figure 5:**
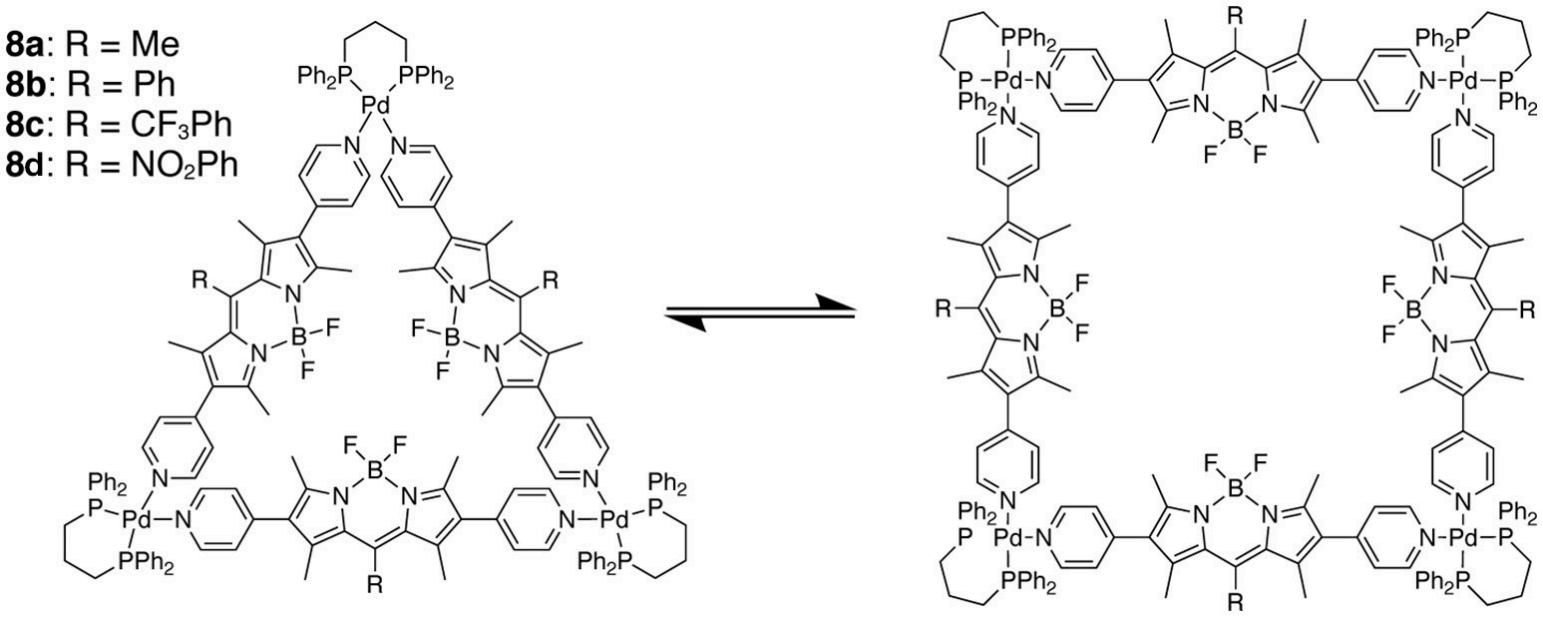
Dynamic equilibrium between the triangular and square forms of macrocycles **8a**–**8d**.

The rigid and planar skeleton of the BODIPYs can act as a linear spacer of the macrocyclic architectures. 4-Pyridyl moieties were introduced at the β positions of a series of BODIPYs to prepare the rod-like bidentate ligands. Self-assembly of the BODIPY ligands and Pd(II) complexes afforded the macrocyclic supramolecules **8a**–**8d** as a mixture of architectures with triangular and square geometries (Figure 5; Gupta et al., 2017a). The two forms of the supramolecules are in equilibrium in solution, and the ratio depends on the solvent. ^1^H NMR spectroscopy suggests that in less polar solvents (dichloromethane and chloroform) both the triangular and square species are present. On the other hand, the use of more polar solvents (acetone, dimethyl sulfoxide, and methanol) produced only the triangular form which is entropically favored. These supramolecules strongly interacted with biomolecules, such as protain and DNA, and were found to be cytotoxic against brain cancer cells. Dipyridyl-appended BODIPYs also coordinate to Ru(II) or Ir(III) ions to produce macrocyclic rectanglar architectures, which exhibit aggregation-induced emission properties and/or a selective citotoxicity (Gupta et al., 2016, 2017b).

**Figure 6:**
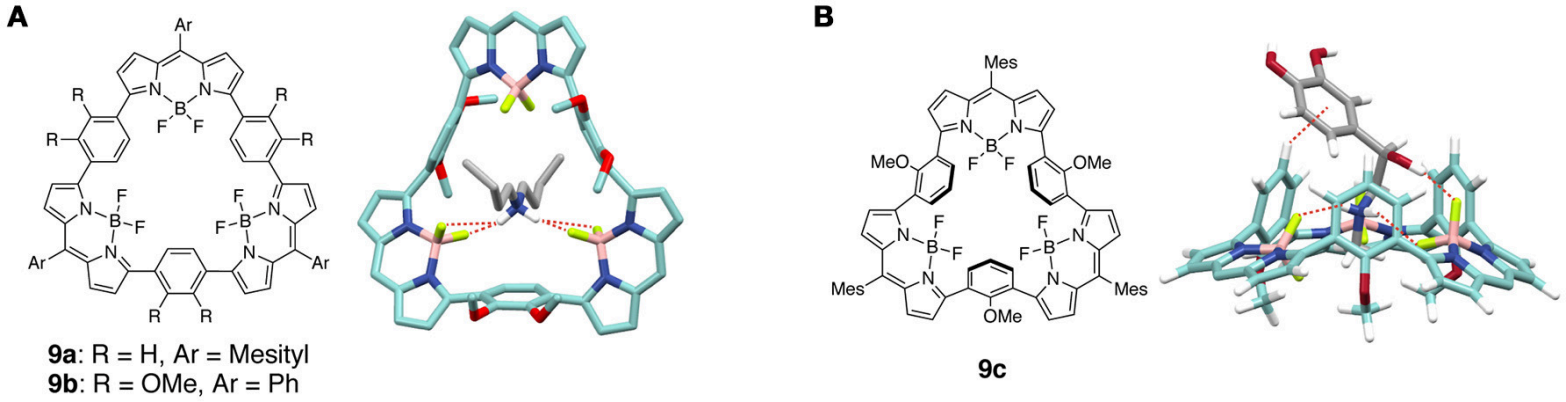
**(A)** (left) Chemical structure of macrocyclic BODIPY trimers **9a,b**. (right) Structure of pseudorotaxane of **9b** and a dibutylammonium cation determined by X-ray crystallography. Hydrogen bonds between fluorine atoms of **9b** and hydrogen atoms of the dibutylammonium cation are shown as dashed lines. Phenyl groups in **9b**, counter anions, hydrogen atoms, and solvent molecules are omitted for clarity. **(B)** (left) Chemical structure of bowl-shaped BODIPY macrocycle **9c**. (right) Structure of pseudorotaxane of **9c** and adrenaline obtained by DFT calculations. Intermolecular interactions between adrenaline and **9c** are shown as dashed lines. Mesityl groups are omitted for clarity. Adapted with permission from Nakamura et al. (2016). Copyright 2016 Wiley-VCH Verlag GmbH & Co. KGaA, Weinheim.

There are some covalently-linked molecular macrocyclic dipyrrin complexes which show a unique molecular or ion recognition ability. For instance, several BODIPY oligomers utilized multiple B–F bonds for molecular recognition. In BODIPY, the large difference in electronegativity between B and F leads to the electronic polarization of each B–F bond. In fact, the fluorine atoms of BODIPY electrostatically interact with cationic guests. A series of macrocyclic BODIPY trimers **9a**–**9c** were designed and synthesized to accumulate the B–F bonds in their cavities (Figure 6) (Sakamoto et al., 2010; Nakamura et al., 2016). The planar macrocycles **9a** and **9b** recognize the dibutylammonium cation to form a pseudorotaxane, which is stabilized by the non-classical BF_2_···H–N hydrogen bonds (Sakamoto et al., 2010). The bowl-shaped trimer **9c** formed a pseudorotaxane via the unidirectional threading of ammonium guests such as the benzylbutylammonium ion and adrenaline (Nakamura et al., 2016). Interestingly, linear BODIPY oligomers selectively capture a cesium cation via a multiple B–F···M^+^ interaction by forming the folded conformations (Sakamoto et al., 2012). A dinuclear aluminum(III) dipyrrin complex also shows the strong recognition of alkaline earth ions (Saikawa et al., 2016) and a cyclic BODIPY trimer captures fullerene in a 1:1 stoichiometry (Ke et al., 2017).

### Self-assembled cage-like architectures

A distinctive advantage of the discrete cage architectures over macrocycles is that they can more strongly and selectively recognize guest molecules within their cavities. This has encouraged chemists to extensively explore the host-guest functionality of cage supramolecules. Several cage architectures comprising dipyrrin complexes have been synthesized and reported as functional materials such as a sensor, host, and photosensitizer.

**Figure 7:**
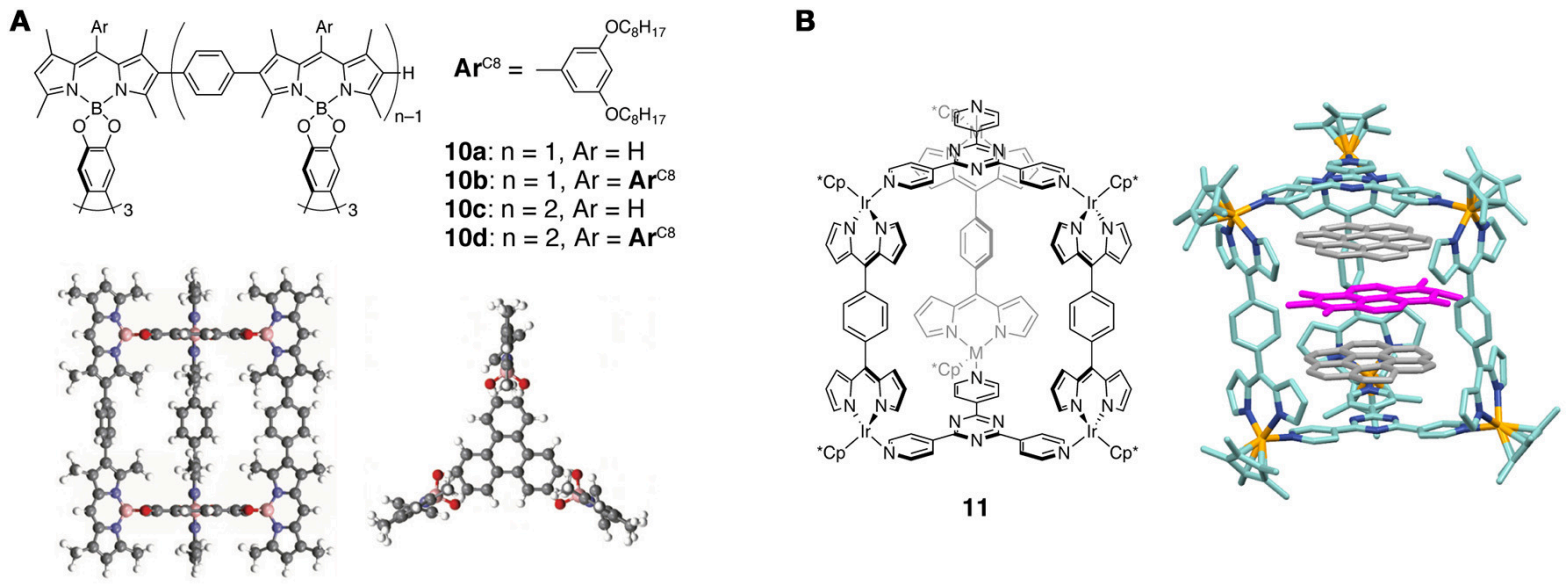
**(A)** (top) Chemical structures of boron-bridged supramolecular cages **10a**–**10d**. (bottom) Structure of **10b** (side and top view) obtained by AM1 calculations. Adapted with permission from Maeda et al. (2013c). Copyright 2013 The Royal Society of Chemistry. **(B)** (left) Chemical structure of Ir(III)-bridged supramolecular cage **11**. (right) Structure of multicomponent host-guest complex of **11**, coronenes, and *N*,*N*'-dimethyl-1,4,5,8-naphthalene-tetracarboxylic diimide determined by X-ray crystallography. Hydrogen atoms are omitted for clarity.

The first dipyrrin-based cage architectures were constructed via the B–O bond formation. Boron-bridged triangular-prism-shaped cages **10a**–**10d** were synthesized from linearly arranged BODIPY oligomers and 2,3,6,7,10,11-hexahydroxytriphenylene (Figure 7A) (Maeda et al., 2013c). The boron centers adopt the tetrahedral coordination geometry and thus keep the triphenylene and dipyrrinate planes orthogonal to each other. A theoretical study suggested that the size of the triangular prism composed of the six boron atoms is 10.02 Å in width and 12.50 Å in height. Fluorescence of the cage compounds was mostly quenched likely due to the photoinduced electron transfer (PeT) from the triphenylene unit to the adjacent dipyrrin unit. The same BODIPY oligomers were also transformed into box- and ladder-shaped architectures using 1,2,4,5-tetrahydroxybenzene.

Another trigonal prismatic supramolecular cage was synthesized using mono(dipyrrinato)iridium(III) complex units. The dinuclear Ir(III) complexes of the linear bis(dipyrrin) ligand reacted with planar tripodal ligands to give the self-assembled cage supramolecule **11** without the assitance of any template (Figure 7B) (Singh et al., 2015). Due to the electron-deficient character of the triazine-centered tripodal ligand, the cage compound quantitatively encapsulates two electron-rich planar guests, i.e., coronenes. In addition, the host-guest complex recognizes an extra electron-deficient guest between the two coronene molecules to form a multi-component 1:2:1 complex. X-ray crystallography revealed that the cage has almost a perfect trigonal prism structure, and the average interplanar distance in the 1:2:1 complex is 3.4–3.5 Å. Both the donor-acceptor nature of the host/guest molecules and perfectly matched cavity size (approximately four times the π-π stacking distance) coutributed to the selective encapsulation of the aromatic molecules.

**Figure 8:**
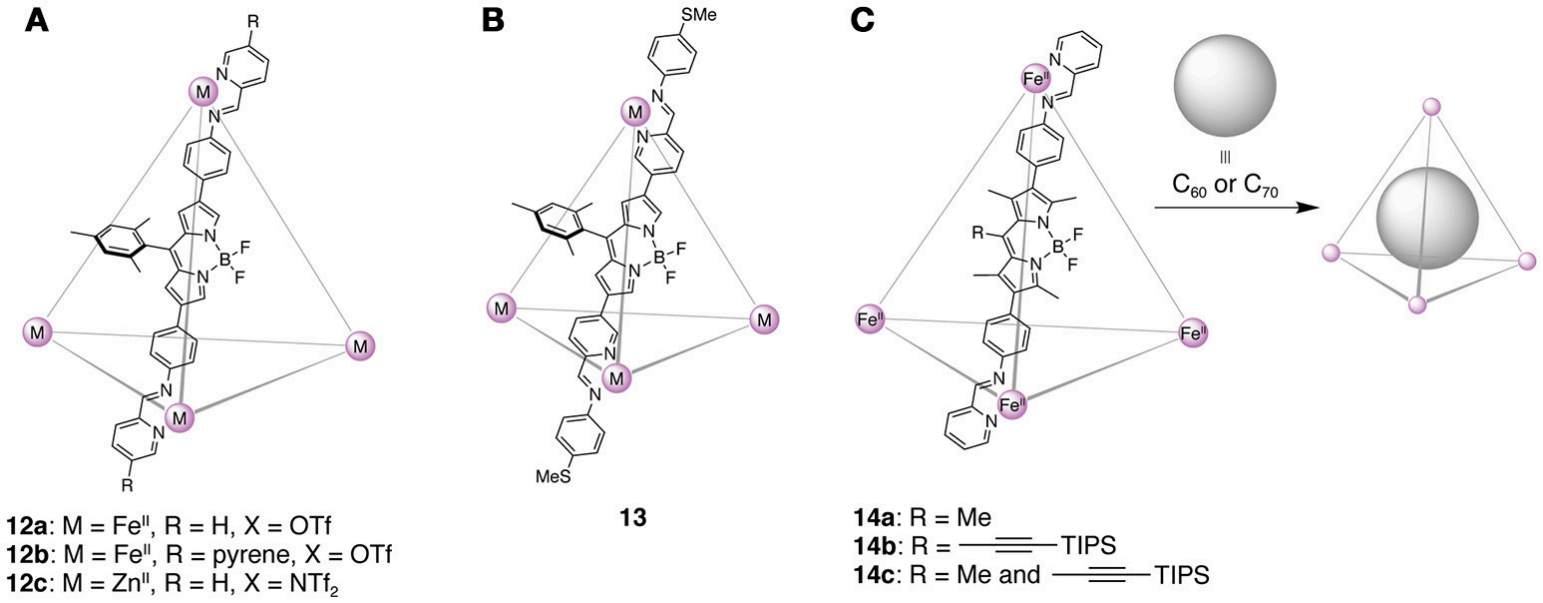
**(A)** Tetrahedral BODIPY cages **12a**–**12c**. The structure of only one edge is shown for clarity. **(B)** Methylthio-substituted tetrahedral cage **13** involved in the autocatalytic system of photooxidation-driven subcomponent exchange reactions. **(C)** Tetrahedral cages **14a**–**14c** and encapsulation of fullerenes.

Nitschke and coworkers constructed a series of self-assembled cage architectures based on BODIPYs via dynamic imine bond formation. They employed a 2-formylpyridyl group as an aldehyde subcomponent of the imines, because self-assembly of the primary amine and 2-formylpyridyne subcomponents with Fe(II) or Zn(II) ions produces an octahedral tris(chelate) complex via the sequential formation of the Schiff base and coordination bond. Therefore, by tethering either the two aldehydes or two amines by a linear linker—β-disubstituted BODIPY—, tetrahedral metal-organic cages were obtained. The first tetrahedral BODIPY cages **12a**–**12c** are composed of linear bis(aminophenyl)BODIPYs and formylpyridine derivatives (Figure 8A) (Neelakandan et al., 2014). ^1^H and ^19^F NMR spectroscopies suggested that the configurations around the metal centers are all Δ or all Λ. Since the tetrahedral cages possess the cationic metal complex moieties, they are capable of recognizing various anions such as acetate and halide. The guest binding leads to changes in the color and fluorescence intensity. The cage **12a** also functioned as a reaction-based indicator for the visual recognition of amino acids as amine sources, because the supramolecular assembly can undergo a subcomponent exchange with the added amines. The exchange reaction resulted in the release of the bis(aminophenyl)BODIPY units that show absorption/emission properties different from those of the cage architecture. Later, the functions of the BODIPY-based metal-organic cages were further developed. An autocatalytic system of photooxidation-driven subcomponent exchange reactions was constructed using the cage **13** composed of bis(formylpyridyl) BODIPY and methylthio-substituted anilines (Figure 8B) (Neelakandan et al., 2015). The BODIPY units acted as a photosensitizer to generate singlet oxygen, which readily oxidized the methylthio groups into sulfoxides. The resultant electron-deficient aniline residues with sulfoxide were then replaced by iodoaniline, which enhanced the photocatalytic activity of the cage and accelerated the subcomponent exchange reactions. In addition, unique photophysical properties of the newly designed BODIPY cages **14a**–**14c** have recently been reported (Figure 8C) (Musser et al., 2017). Due to the extended π-conjugation along the edge of the tetrahedral cage, strong excitonic interactions between the neighboring BODIPY chromophores took place. As a result, an initial emissive excited state rapidly relaxed to a delocalized nonemissive state, which was then changed to a geometrically-relaxed state. On the other hand, upon the encapsulation of fullerene, fast electron transfer from the cage to fullerene occured in the initial photoexcited state.

## Infinite and periodic supramolecular architectures and their applications to materials

Reversible bonds are useful for constructing a variety of infinite supramolecular architectures as well as the discrete counterpart. In particular, coordination-driven infinite self-assemblies (*i.e*., coordination polymers) have been intensively studied because their polymeric and periodic structures are well designed and synthesized. In addition, the functions obtained from the coordination polymers are often different from those of discrete architectures and applicable to materials science. In this section, the metal-ion-mediated self-assembly of 1D polymers, 2D nanosheets, and 3D porous networks based on the dipyrrin complexes are introduced.

### One-dimensional coordination polymers

**Figure 9:**
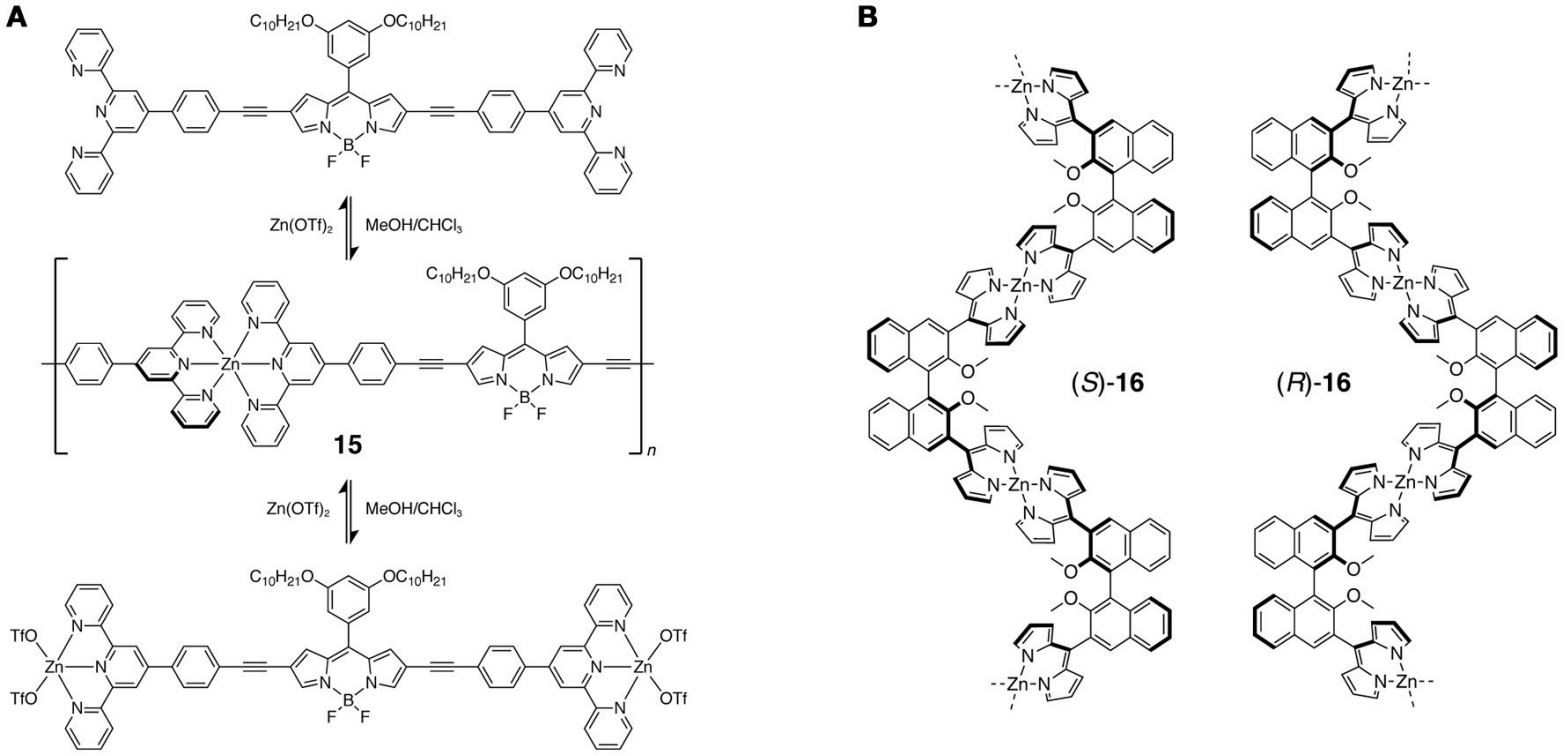
**(A)** Dynamic equilibrium between a bis(terpyridyl)BODIPY ligand, 1D coordination polymer **15**, and a monomeric complex upon addition of Zn(OTf)_2_ in MeOH/CHCl_3_ mixture. **(B)** Chiroptical bis(dipyrrinato)zinc(II) one-dimensional coordination polymers (*S*)- and (*R*)-**16**.

A 1D coordination polymer containing fluorescent BODIPY units exhibited a unique dynamic equilibrium between the polymeric and monomeric state. The β positions of BODIPY was modified with (ethynylphenyl)terpyridyl groups so that the octahedral coordination of the terpyridyl ligands with Zn(II) ions produced a one-dimensional polymer **15** (Figure 9A) (Bozdemir et al., 2009). Upon the addition of the Zn(II) ion, ^1^H NMR signals of the ligand were broadened, indicating the formation of the coordination polymer. The broadness of the signal was maximal at the 1:2 ratio of Zn(II) ion to the ligand. With an excess amount of Zn(II) ion, the ^1^H NMR signal was sharpened again due to dissociation of the polymer and the formation of mono(terpyridyl)zinc(II) complex structures. Both the ligand and coordination polymer show a strong absorption and intense fluorescence in 80:20 CHCl_3_/MeOH (fluorescence quantum yield, φ_F_ = 0.47 for the ligand, 0.49 for the coordination polymer). Similar 1D coordination polymers were synthesized using Fe(II) ions although their emission was quenched.

One-dimensional coordination polymers with bis(dipyrrinato)metal(II) complexes exhibited various photofunctions. Dipyrrin dimers that are obtained by tethering the two dipyrrin units at the *meso* positions can form oligomer or polymer structures upon tetrahedral coordination with divalent metal ions (Maeda et al., 2006; Miao et al., 2009; Matsuoka et al., 2015). Recently, chiroptical one-dimensional coordination polymers (*R*)- and (*S*)-**16** comprised of the bis(dipyrrinato)zinc(II) complex units were reported (Figure 9B) (Aoki et al., 2017). The chiral ligand is composed of two dipyrrin ligands bridged by the binaphthyl moiety. Each enantiomer of the ligand reacted with Zn(II) acetate to produce homochiral coordination polymers. They can be exfoliated into single polymer chains upon ultrasonication in an organic solvent, retaining their polymeric nature with a length of up to 3.19 μm. The dispersed polymers show a circularly polarized luminescence (CPL), which indicates that the chirality is transferred from the chiral binapthyl moieties to the luminescent bis(dipyrrinato)zinc(II) complex moieties. Interestingly, the CPL intensity was 5.9 times greater than that of the corresponding monomeric complex. It is suggested that the steric hindrance between the binapthyl and dipyrrin moieties is responsible for the chirality transfer, and that the enhancement of the CPL activity may originate from the suppressed thermal fluctuation of this steric hindrance.

A variety of 1D coordination polymers featuring dipyrrin complexes have been found in the crystalline solid state (Halper et al., 2004; Kilduff et al., 2010; Pogozhev et al., 2010; Béziau et al., 2012a, 2014; Mazel et al., 2017). For example, heteroleptic mono(dipyrrinato)copper(II) complexes with the acetylacetonato-type ancillary ligands were employed as building units (Halper et al., 2004; Kilduff et al., 2010; Pogozhev et al., 2010; Béziau et al., 2012a). Either the dipyrrin or acetylacetonato-type ligand was connected to an additional coordinating moiety such as the pyridyl and cyano group. This moiety further coordinated to the Cu(II) ion of the adjacent mono(dipyrrinato)copper(II) complex to give self-assembled 1D infinite coordination networks.

### Two-dimensional coordination nanosheets

**Figure 10:**
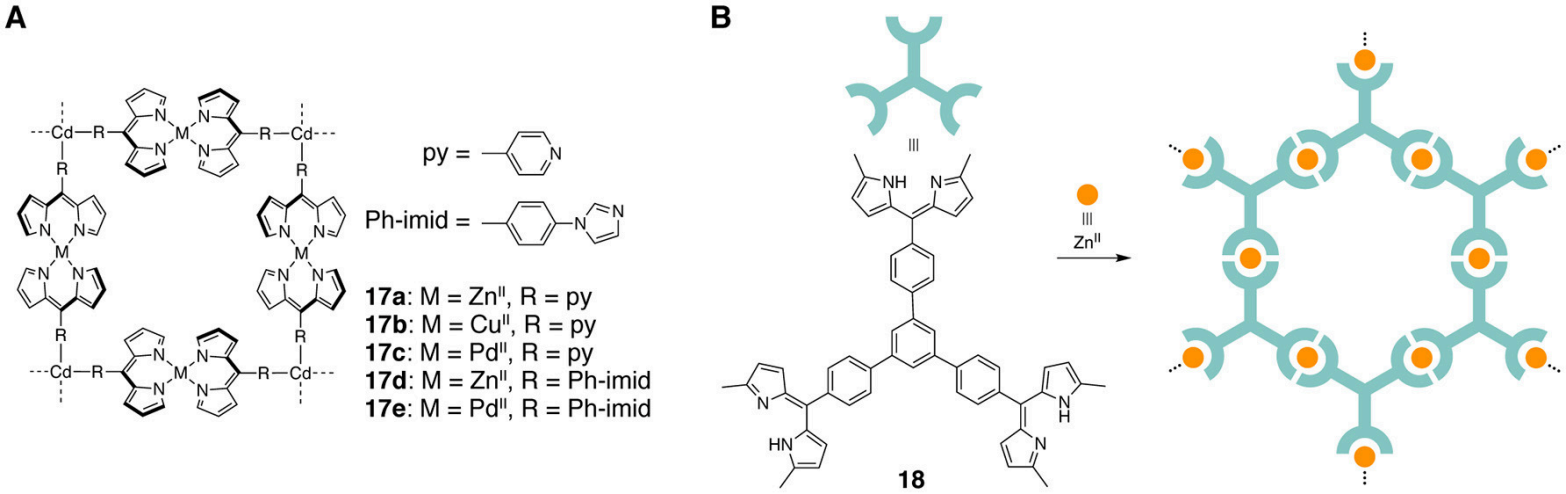
**(A)** 2D grid-type coordination polymers **17a**–**17e** based on bis(dipyrrinato)metal(II) complexes bearing heterocyclic units. **(B)** Schematic illustration showing the synthesis of photofunctional bis(dipyrrinato)zinc(II) complex nanosheet from **18** and Zn(II) ions.

A series of 2D grid-type coordination polymers based on bis(dipyrrinato)metal(II) complexes were obtained as a crystalline state (Béziau et al., 2012b, 2013b). The dipyrrin ligands were modified with an additional coordinating moiety (pyridyl or imidazolyl group). The dipyrrin and additional group coordinated to different metal ions because of the difference in their denticity and charge. This selective coordination resulted in the formation of two-dimensional coordination polymers **17a**–**17e** (Figure 10A) (Béziau et al., 2013b). Interestingly, either a sequential complexation or one-pot reaction resulted in the formation of the same coordination networks. In addition, the shape of the two-dimensional grids and their packing arrangement depend on the metal ions, ligands, and solvents incorporated in the crystal. For instance, the Zn(II)-Cd(II) polymer **17d** is stacked with an offset, whereas the Pd(II)-Cd(II) polymer **17e** is arranged to form a 3-fold interpenetrated structure. The coordination polymers comprising Zn(II) and Cd(II) ions were weakly luminescent in the crystalline state.

A novel synthetic strategy for two-dimensional nanosheets featuring bis(dipyrrinato)zinc(II) complex units was recently achieved. The reaction between a three-way tris(dipyrrin) ligand **18** and Zn(II) salt at an oil/liquid interface gave multi-layer nanosheets, whereas the reaction at an air/liquid interface produced single- or few-layer nanosheets (Figure 10B) (Sakamoto et al., 2015a). The liquid interfaces acted as flat reaction fields that suppressed the random aggregation of the coordination networks. The sheet transferred on a transparent electrode functioned as a photoactive layer in a photoelectric conversion system. The nanoporous nature of the periodic two-dimensional networks is presumably responsible for the better photosensitizing ability than analogs not forming periodic self-assembled monolayers. Later similar types of micro- and nanosheets containing zinc(II) porphyrin moieties were prepared (Sakamoto et al., 2017). The obtained sheets absorbed a broad range of visible light (400–650 nm) and exhibited a 2-fold better photoelectric conversion ability than the nanosheet composed of **18**.

### Three-dimensional porous coordination networks

A number of porous coordination networks (i.e., metal-organic frameworks, MOFs) bearing dipyrrin complexes have been developed and investigated. The dipyrrin ligands or BODIPYs that are connected to other coordination units serve as a multitopic ligand to form various self-assembled 3D networks (Béziau et al., 2013c, 2015; Zhou et al., 2013; Li et al., 2015).

**Figure 11:**
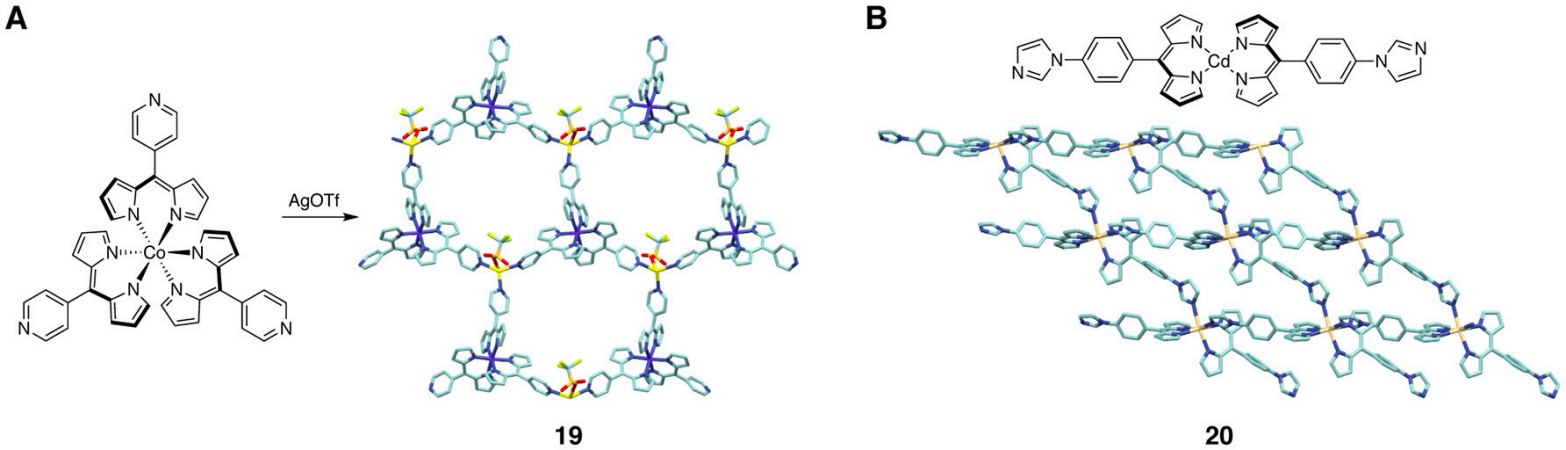
**(A)** Crystal structure of self-assembled MOF **19** based on tris(dipyrrinato)cobalt(III) metalloligand and Ag(OTf). **(B)** Crystal structure of luminescent MOF **20** consisting of the imidazolyl-appended bis(dipyrrinato)cadmium(II) complex.

Cohen and coworkers synthesized a wide variety of MOFs comprised of tris(dipyrrinato)metal(III) complex units (metal = cobalt, iron, gallium, and indium) (Halper and Cohen, 2005; Murphy et al., 2005; Halper et al., 2006; Garibay et al., 2007; Stork et al., 2007). The dipyrrin ligands were modified with extra coordinating groups at the *meso* position, and the corresponding tris(dipyrrinato)metal(III) complexes thus serves as tripodand “metalloligands.” These building blocks with a 3-fold symmetry undergo a second complexation to another metal center to afford periodic MOF structures such as **19** (Figure 11A) (Halper and Cohen, 2005). The second ligating moieties involved cyano, pyridyl, quinolinyl, and carboxyl groups, and the second metal center was either the Ag(I) or Zn(II) ion. In particular, the MOF composed of a cyanophenyl-appended dipyrrin ligand was robust to solvent removal, and adsorbed various aromatic guest molecules. In addition, the MOF more strongly adsorbed nitroaromatic compounds than toluene.

Hosseini and coworkers developed luminescent MOFs based on cadmium(II) dipyrrin complexes (Béziau et al., 2013a). Unlike the zinc(II) analogs, the metal centers in the bis(dipyrrinato)cadmium(II) complexes can adopt five or six coordination numbers in the presence of additional coordinating groups. An imidazolyl-appended dippyrrin ligand reacted with Cd(II) ions to form a 3D periodic network **20**, in which the metal centers were coordinated by two dipyrrinate ligands and two imidazolyl moieties (Figure 11B). In a similar way, a pyridyl-substituted dipyrrin ligand formed coordination polymers with Cd(II) ions; but in this case, the coordination network is one-dimensional or two-dimensional. All the crystalline MOFs showed a ligand-centered luminescence around 600 nm.

**Figure 12:**
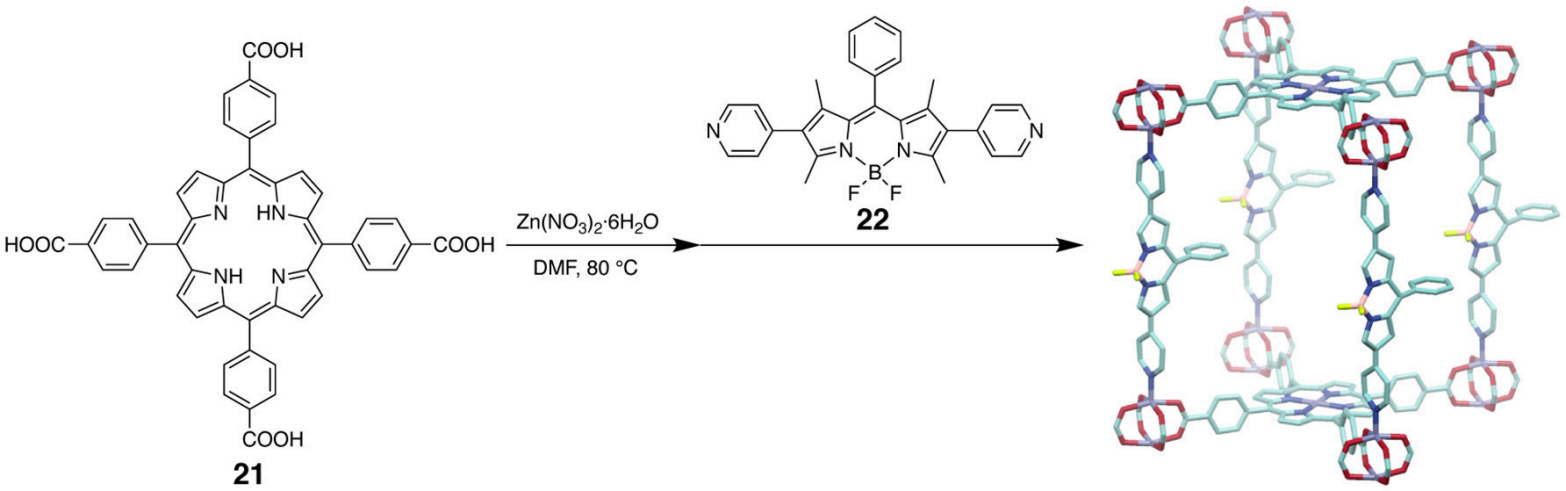
Schematic illustration showing the synthesis of a pillared-paddlewheel type MOF through reaction of **21** with Zn(II) ions followed by reaction with BODIPY **22**. The structure of the MOF is determined by X-ray crystallography. Hydrogen atoms and disordered solvents are omitted for clarity.

An MOF possessing both BODIPY and metalloporphyrin struts showed cooperative light-harvesting properties (Lee et al., 2011). The nearly black, pillared-paddlewheel type MOF was synthesized from tetracarboxylic porphyrin **21**, bipyridyl-functionalized BODIPY **22**, and Zn(II) ions (Figure 12). A control MOF material comprised of a non-chromophoric strut in place of the porphyrin ligand showed a green solid-state luminescence from the BODIPY moieties. In the MOF with porphyrin, on the other hand, efficient energy transfer from the BODIPY to the porphyrin units occurred upon photoexcitation, and the emission was observed from only the Zn(II) porphyrin units. Therefore, the BODIPY struts served as antenna chromophores for the excitation of the porphyrinic chromophores. The MOF with the porphyrin and BODIPY units is capable of harvesting light across the entire visible spectrum.

### Other supramolecular infinite structures

There are a few examples of dipyrrin-based infinite supramolecular architectures constructed via noncovalent interactions other than coordination bonds (Salazar-Mendoza et al., 2007; Telfer and Wuest, 2007, 2009; Chen et al., 2017). For example, hydrogen-bonded supramolecular networks were constructed as crystalline solids from the tris(dipyrrinato)cobalt(III) complexes possessing carboxyl or diaminotriazinyl groups (Telfer and Wuest, 2007, 2009). Another example is that an amphiphilic aza-BODIPY dye formed two different *J*-aggregate structures (nanoparticles and nanorods) in a competing self-assembly process (Chen et al., 2017). These two aggregates exhibit distinct near-infrared optical properties.

## Conclusion

Studies of supramoleular architectures created via the dynamic reversible bond formation have significantly progressed to afford more elaborate structures and functions. In order to realize sophisticated novel functions of the supramolecular self-assemblies, the dipyrrin complexes have been employed as a functional unit because of their outstanding properties and functions such as a high photostability and strong light absorption/emission. We described the recent developments in supramolecular architectures comprising the dipyrrin complexes, focusing on their construction via metal-ligand coordination and dynamic covalent bond formation, and on their unique functions. Although the early studies concerned only the formation of the self-assemblies as a discrete supramolecule, various artificial supramolecules such as helicates, macrocycles, and cages with unique functions have recently been reported. Dipyrrin complexes are also incorporated into the infinite supramolecular architectures, polymers, sheets, and porous materials as 1D, 2D, and 3D architectures, respectively. These architectures can be applied to functional materials with the advantage of the polymeric and periodic nature of the infinite structures.

If the dipyrrin-complex units in supramolecular self-assemblies communicate with each other, cooperative functions and enhanced response to an external stimuli are expected. Consequently, supramolecular architectures bearing the dipyrrin complexes would lead to a wide variety of materials for sensing, imaging, catalysis, photodynamic therapy, and energy conversion.

## Author contributions

All authors listed have made a substantial, direct and intellectual contribution to the work, and approved it for publication.

### Conflict of interest statement

The authors declare that the research was conducted in the absence of any commercial or financial relationships that could be construed as a potential conflict of interest.
